# Exploration of the Synergistic Regulation Mechanism in Cerebral Ganglion and Heart of *Eriocheir sinensis* on Energy Metabolism and Antioxidant Homeostasis Maintenance under Alkalinity Stress

**DOI:** 10.3390/antiox13080986

**Published:** 2024-08-14

**Authors:** Meiyao Wang, Jun Zhou, Gangchun Xu, Yongkai Tang

**Affiliations:** 1 Wuxi Fisheries College, Nanjing Agricultural University, Wuxi 214081, China; wangmy@ffrc.cn; 2 Key Laboratory of Freshwater Fisheries and Germplasm Resources Utilization, Ministry of Agriculture and Rural Affairs, Freshwater Fisheries Research Center, Chinese Academy of Fishery Sciences, Wuxi 214081, China; 3 Freshwater Fisheries Research Institute of Jiangsu Province, Nanjing 210017, China; finedrizzle@163.com

**Keywords:** *Eriocheir sinensis*, alkalinity stress, cerebral ganglion, heart, synergistic regulation

## Abstract

(1) The development and utilization of the vast saline–alkali land worldwide is an important way to solve the worsening food crisis. *Eriocheir sinensis*, due to its strong osmotic regulation capability and its characteristics of being suitable for culturing in alkaline water, has become a potential aquaculture species in saline–alkali water. The brain and heart are the key tissues for signal transduction and energy supply under environmental stress. (2) This study is the first to explore the synergistic regulatory molecular mechanism by integrated analysis on cerebral ganglion proteomics and heart metabolomics of *Eriocheir sinensis* under alkalinity stress. (3) The results indicate that the cerebral ganglion and heart of *E. sinensis* were closely related in response to acute alkalinity stress. The differential regulatory pathways mainly involved regulation of energy metabolism, amino acid metabolism, and homeostasis maintenance. Importantly, alkalinity stress induced the regulation of antioxidants and further adjusted longevity and rhythm in the cerebral ganglion and heart, reflecting that the cerebral ganglion and heart may be the key tissues for the survival of *Eriocheir sinensis* under an alkalinity environment. (4) This study provides a theoretical reference for research on the regulation mechanism of *E. sinensis* under alkalinity condition and contributes to the development of aquaculture in saline–alkali water.

## 1. Introduction

In recent years, saline–alkali land worldwide has continuously expanded due to human activities, including deforestation and improper fertilization, etc. The term alkalinity typically refers to the collective concentration of substances capable of neutralizing and reacting with strong acids within water. In general, the total alkalinity of water primarily consists of bicarbonate (HCO_3_^−^) and carbonate (CO_3_^2−^) ions, collectively referred to as carbonate alkalinity [1]. Therefore, the cultivation of saline–alkali-tolerant crops and aquaculture varieties, as well as the vigorous development and utilization of the vast saline–alkali land, have reached an agreement by governments worldwide. *Eriocheir sinensis* (*E. sinensis*), belonging to Class Crustacea, Genus Eriocheir, is an indigenous species in East Asia. Due to its euryhaline and strong migration ability, *Eriocheir sinensis* is widely distributed in the world. It has become an important economic aquaculture species due to its delicacy and rich nutrition [2,3,4]. As a catadromous migration aquatic species, *E. sinensis* has good osmoregulation ability. Moreover, proper pH for its living is alkaline (pH ranges from 7.5 to 9.5) [5]. In addition, it has been reported that the meat quality of *E. sinensis* cultured in saline–alkaline water can be improved. Therefore, *E. sinensis* is regarded as a suitable aquatic species for cultivation in saline–alkaline water [6].

Current studies on the physiological response mechanism of shrimp to alkalinity mainly involve the regulation mode of alkalinity on histological and omics in the gill and hepatopancreas, the cloning of the key anti-alkalinity stress regulatory gene such as carbonic anhydrase and HSP40 [7,8,9,10,11,12]. There are few studies on the regulation mechanism in crabs to alkalinity stress. The research on gill metabolomics of *E. sinensis* under alkalinity stress manifested that the anti-inflammatory system was activated to make an active response to stress [13]. The research on alkalinity stress in the model organism zebrafish (*Danio rerio*) primarily focused on investigating the impact of alkalinity on a toxic substance, such as peracetic acid, and its subsequent effect on fish development [14].

The brain is the regulation center of stress response to various environmental factors, including osmotic pressure, temperature, dissolved oxygen content, etc. [15,16,17]. The heart plays a key regulatory role in power supply under the control of the central nervous system. In the past ten years, there have been few studies on the regulation of crab hearts. Proteomics systematically reveal the molecular network that controls complex life activities at the protein level [18]. Metabolomics, which is closest to the biological phenotype, is a good method to reveal the regulatory mechanisms of biomolecules [19].

In view of the important regulatory role of brain and heart in the response to adverse environmental factors, in this study, the molecular mechanism of co-regulation of cerebral ganglion proteomics and heart metabolomics of *E. sinensis* under alkalinity stress are carried out. Antioxidant capacity is an important regulatory factor for cardiac metabolism. Therefore, the antioxidant response of *E. sinensis* under alkalinity is also discussed. This study not only reveals respective response patterns to alkalinity stress in the cerebral ganglion and heart of *E. sinensis*, but also represents their co-regulatory pathways and synergistic regulation mechanism in resistance to alkalinity stress. This study provides a theoretical reference for research on the regulation mechanism of *E. sinensis* under saline–alkali conditions; it also provides a theoretical guidance on the cultivation of new varieties of saline–alkali-tolerant *E. sinensis* and other resistant crustaceans and contributes to the development of aquaculture in saline–alkali water.

## 2. Materials and Methods

### 2.1. Experimental Crabs and Alkalinity Stress

*Eriocheir sinensis* for experiment were collected from breeding farm of Haorun Group (Taizhou, China). Healthy *E. sinensis* with neat size and complete appendages were selected (average weight was 54.48 ± 3.13 g). Diets special for *E. sinensis* were fed at 9 am and 2 pm every day during the acclimation period. The feeding ratio was 5% of the crab’s weight. The water was continuously aerated and renewed by half per day. The residual feed and excrement were removed once a day, water quality was monitored each day: water temperature was maintained at (21 ± 1) °C, pH was 7.8, and the concentrations of NO_2_^−^ and NH_3_-N were 0.005 mg/L and 0.1 mg/L, respectively. The concentration of DO was 6 mg/L. Feedings were stopped the day before the experiment. In this study, the control group and alkalinity group were set up, and each group contained five parallel groups. Bicarbonate was added to achieve alkalinity of 60 mmol/L. The total alkalinity was measured using the methyl orange hydrochloride calibration method. After the alkalinity treatment lasted for 6 h, two *E. sinensis* (one female and one male) were randomly selected from each parallel group of the control and the experimental group, respectively. The hearts and cerebral ganglion were sampled. The hearts of a male and female *E. sinensis* in the same parallel group were mixed as one sample. Finally, five heart samples were obtained and quickly frozen in liquid nitrogen and then transferred to the −80 °C refrigerator for subsequent metabolomics analysis. With the same method, three cerebral ganglion samples were obtained.

### 2.2. Measurement of Serum Biochemical Parameters

As previously mentioned, when we collected tissue for omics analysis, we also collected blood to prepare serum for the determination of biochemical parameters, including SOD (superoxide dismutase), GSH-Px (glutathione peroxidase), T-AOC (total antioxidant capacity), and MDA (malondialdehyde). We used a syringe to collect blood from the base of the fourth periopod of *E. sinensis*, added the same amount of anticoagulant (acid citrate-dextrose), centrifuged it at 4 °C for 10 min at 4000 r/min, and collected the supernatant. Serum biochemical parameters were measured using kits manufactured by Nanjing Jiancheng Bioengineering Institute (Nanjing, China).

### 2.3. Proteomics Analysis on Cerebral Ganglion of E. sinensis

#### 2.3.1. Proteins Extraction, Separation, Enzymolysis, and Desalination

Tissues were homogenized in liquid nitrogen, the lysis solution and protease inhibitor PMSF were added to reach the final concentration of 1mM, and then the solution was ultrasonic-crushed on ice. The solution was centrifuged at room temperature for 10 min (12,000 rpm), the supernatant was collected, and then centrifuged again to obtain the supernatant, which was the total protein solution of the sample. The protein concentration was determined using the BCA method and stored at −80 °C. Ten microgram protein was taken from each sample and separated by 12% SDS-PAGE. The gel was stained using Coomassie brilliant blue using the eStain LG protein staining instrument. The stained gel was scanned by an automatic digital gel imaging and analysis system. According to the protein concentration, the appropriate amount of protein was taken from each sample, and the samples in different groups were diluted and adjusted to the same concentration with the lysis solution. DTT and iodoacetamide were added successively, and finally, acetone was added for protein precipitation. The precipitation was collected after centrifugation at 4 °C for 10 min (8000 rpm). Thereafter, NH_4_HCO_3_ was added for precipitation re-dissolution, and Trypsin-TPCK was added and then digested at 37 °C overnight.

After enzymolysis, the peptides were desalted using a SOLA™ SPE 96-well plate. Methanol and pure water (containing 0.1% methanoic acid) were added for column activation, the sample was loaded, and then 0.1% methanoic acid–water was added for washing. Finally, 50% acetonitrile–water was used for elution (containing 0.1% methanoic acid).

#### 2.3.2. LC-MS/MS Analysis and Database Query

The samples were loaded to the 25 cm C18 column (RP-C18, ionopticks) at a flow rate of 300 nL/min, and then made via gradient elution. Mobile phase A: H_2_O-FA (99.9:0.1, *v*/*v*), mobile phase B: ACN-H_2_O-FA (80:19.9:0.1, *v*/*v*/*v*). Gradient elution procedures: 0~66 min, 3–27% B; 66–73 min, 27–46% B; 73–84 min, 46–100% B; 84–90 min, 100% B. The MS procedures: the capillary voltage was 1.4 KV, the drying air temperature was 180 °C, the drying gas flow rate was 3.0 L/min, and the scanning range was 100–1700 *m*/*z*.

The LC-MS/MS original files were imported into MaxQuant (version 1.6.17.0) (Max Planck Institute, Münster, Germany,) for database querying. The search engine used was Andromeda for LFQ non-standard quantitative analysis. To prevent peak mismatch, the search standard was controlled by a false discovery rate (FDR < 0.01). The specific database search parameters were set as follows: the missed cleavage was 2; the fixed modification was carbamidomethyl (C); the variable modification was oxidation (M); the decoy database pattern was reverse; the enzyme was trypsin; the first search peptide tolerance was 20 ppm; and the main search peptide tolerance was 10 ppm.

#### 2.3.3. Gene Ontology (GO) Annotation on Proteomic

Species proteins were used as the background list, and the obtained total protein list was used as the candidate list. A hypergeometric distribution test was used to calculate the *p*-value, representing whether the functional set was significantly enriched in the differential protein list, and then the p-value was corrected by Benjamini and Hochberg’s multiple tests to obtain the FDR. Identified proteins were annotated with Blast2GO (Biobam, Valencia, Spain) [20].

#### 2.3.4. Enrichment Analysis on Differentially Expressed Proteins (DEPs)

Log2(Foldchange) was used to evaluate the expression change ratio of a protein between the experimental group and the control group. The *p*-value calculated via t-test represented the significant difference. The screening conditions were Foldchange > 1.5 or Foldchange < 1/1.5 and *p*-value < 0.05 [21]. The top20 Kyoto Encyclopedia of Genes and Genomes (KEGG) pathways were obtained according to the following conditions: the pathways with gene number in each pathway (listhit) were larger than one were selected, and then the top 20 KEGG pathways were obtained in descending order according to −log10 *p*-value of the selected pathways.

### 2.4. Metabolome Analysis on Heart of E. sinensis 

#### 2.4.1. Extraction of Metabolites from Heart of *E. sinensis* and UPLC-MS Analysis

QC samples, an equal volume mixed sample of each sample, were prepared for the balancing LC/MS system, monitoring the status of instruments and assessing stability of the system during the whole experiment process. Meanwhile, a blank sample was set (53% methanol replaced of sample), which was mainly used to remove background interference. The extraction procedures were as follows: 100 mg samples homogenized in liquid nitrogen were placed in an EP tube, and 500 μL 80% methanol was added. The samples were placed under an ice bath for 5 min after vortexing, and then were centrifuged at 4 °C for 20 min (15,000 rpm). Some supernatant was collected and diluted with pure water and then centrifuged at 4 °C for 20 min (15,000 rpm). Thereafter, the supernatant was collected and loaded for LC-MS analysis [22]. The samples were injected into Vanquish UHPLC (Thermo Fisher, Waltham, MA, USA). The column was HypesilGoldcolumn (C18), with a column temperature of 40 °C and a flow rate of 0.2 mL/min. In the positive-ion mode, mobile phase A was 0.1% methanoic acid and mobile phase B was methanol. In the negative-ion mode, mobile phase A was 5 mM ammonium acetate, pH was 9.0, and mobile phase B was methanol. The gradient elution procedure is shown below: 0–1.5 min, 98%A and 2%B, 1.5–3 min, 15%A and 85%B, 3–10 min, 100%B, 10–12 min, 98%A and 2%B. The specific parameters were set as follows: the scanning range was *m*/*z* 100–1500. The ESI source settings were as follows: spray voltage was 3.5 kV, sheath gas flow rate was 35 psi, aux gas flow rate was 10 L/min, capillary temperature was 320 °C, S-lens RF level was 60, aux gas heater temperature was 350 °C, and MS/MS second-level scanning were data-dependent scans. 

#### 2.4.2. PCA and Partial Least-Squares Discrimination Analysis (PLS-DA) on Differentially Expressed Metabolites (DEMs) 

PCA was used to represent the overall distribution trend in samples between the control and the experiment groups. PLS-DA was used to test whether the model was “over-fitting” [23]. The test procedures were as follows: group marks of the samples were randomly disrupted, and then modelling and prediction were conducted. Each model corresponds to a set of R2 and Q2 values, and their regression lines can be obtained according to the Q2 and R2 values after 200 instances of disruption and modeling. When R2 is larger than Q2 and the intercept between Q2 regression line and Y-axis is less than 0, it is indicated that the model is not “over-fitting” [24].

#### 2.4.3. KEGG Enrichment Analysis on DEMs 

The DEMs were screened in accordance with foldchange and *p*-value, and metabolites with Foldchange > 1.5 or Foldchange < 1/1.5 and *p*-value < 0.05 were selected as DEMs. N is the number of total metabolites participating in KEGG metabolic pathways; n is the number of DEMs in N; y is the number of metabolites annotated to a KEGG pathway; x is the number of DEMs enriched in a KEGG pathway; the ratio condition is x/n > y/N; the pathways which met this ratio condition and *p*-value < 0.05 were set as significantly differentially expressed KEGG pathways; and then the top20 KEGG pathways were obtained.

### 2.5. Combined Analysis on Cerebral Ganglion Proteomic and Heart Metabolome 

All the DEPs and DEMs were mapped to the KEGG database, respectively, then their common pathways which the DEPs and DEMs were both involved in were determined as co-regulatory pathways of both omics.

## 3. Results

### 3.1. Analysis on Serum Biochemical Parameters of E. sinensis under Alkalinity Stress

**Figure 1:**
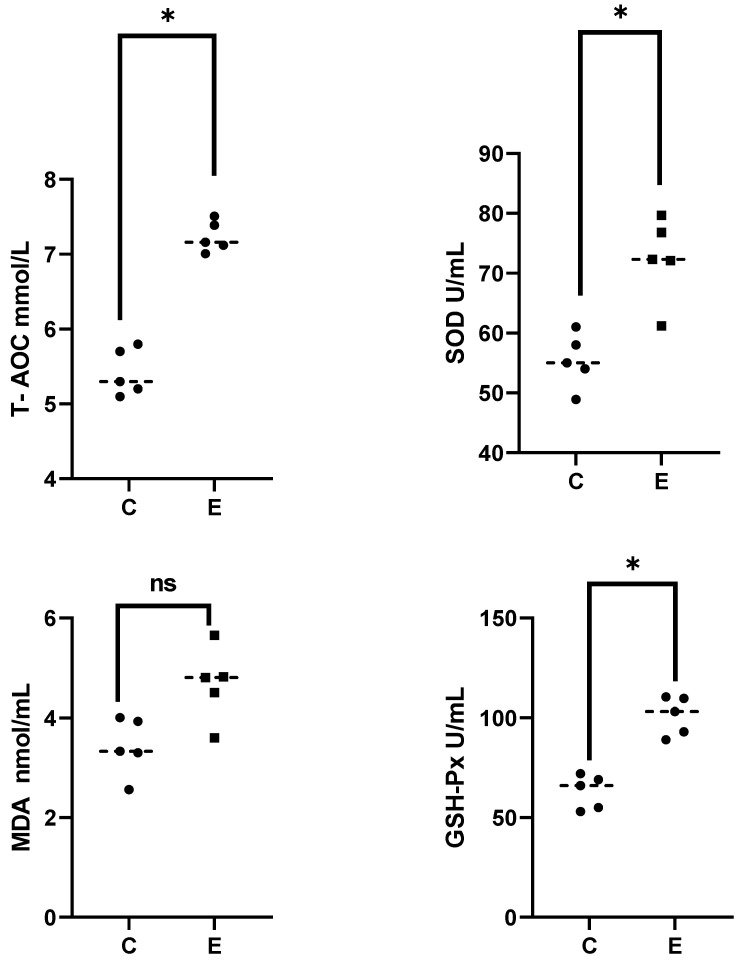
Analysis of serum biochemical parameters in this study. “C” and “E” indicate the control and the experimental group, respectively. The asterisks indicates significant difference. “ns” represents nonsignificant difference.

As depicted in Figure 1, the activity of SOD and GSH-Px, and level of T-AOC in serum were significantly upregulated at 6 h after alkalinity stress (*p* < 0.05), while the content of MDA showed a slight increase but was not significant (*p* > 0.05). Overall, the antioxidant system of *E. sinensis* exhibited an upregulation under acute alkalinity stress.

### 3.2. Proteomic Analysis on Cerebral Ganglion under Alkalinity Stress

**Figure 2:**
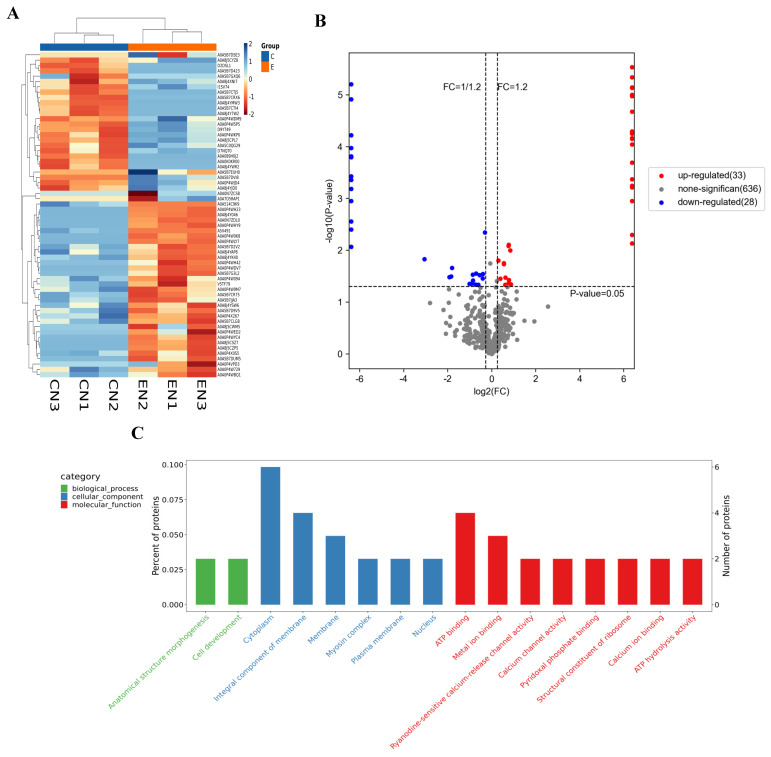

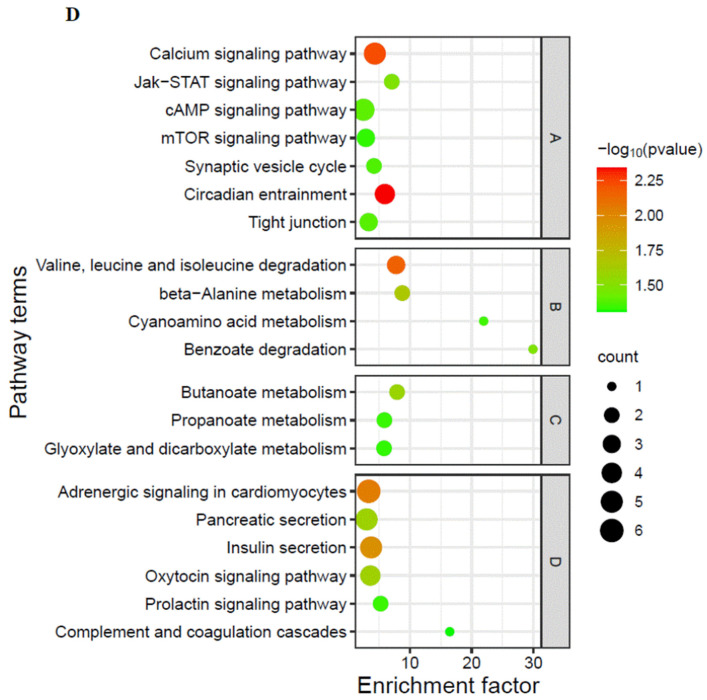
Analysis on proteome in cerebral ganglion of *E. sinensis* induced by alkalinity stress. (**A**). Heatmap of DEPs after alkalinity stress. CN1-CN3 represents the control group, EN1-EN3 represents the alkalinity-stressed group. Rows represented DEPs. (**B**). Volcano plot of DEPs after alkalinity stress. (**C**). TOP30 GO enrichment analysis on DEPs after alkalinity stress. (**D**). Top20 KEGG enrichment analysis on DEPs after alkalinity stress. “A–D” represents four functional categories that the pathways can be classified into: A, signal transduction; B, amino acid metabolism; C, energy metabolism; D; organismal system.

As shown in Figure 2A,B, a total of 61 DEPs were detected after alkalinity stress. GO enrichment analysis of DEPs showed that the items in the Cellular Component subcategory mainly related to cell membranes. The items enriched in the molecular function subclass mainly involved calcium channel activity and regulation of energy metabolism (Figure 2C). 

As shown in Figure 2D, Top20 KEGG pathways were mainly divided into four categories: amino acid metabolism, signal transduction, energy metabolism, and organismal system. Signal transduction pathways involved environment adaptation and antioxidant regulation (mammalian target of rapamycin (mTOR) signaling pathway), homeostasis maintenance (Circadian entrainment), and information presentation (Synaptic vesicle cycle, Tight junction, Calcium signaling pathway). Organismal system pathways involved circulatory system, digestive system, reproductive system, and immune system. 

### 3.3. Heart Metabolomics Analysis under Alkalinity Stress

**Figure 3:**
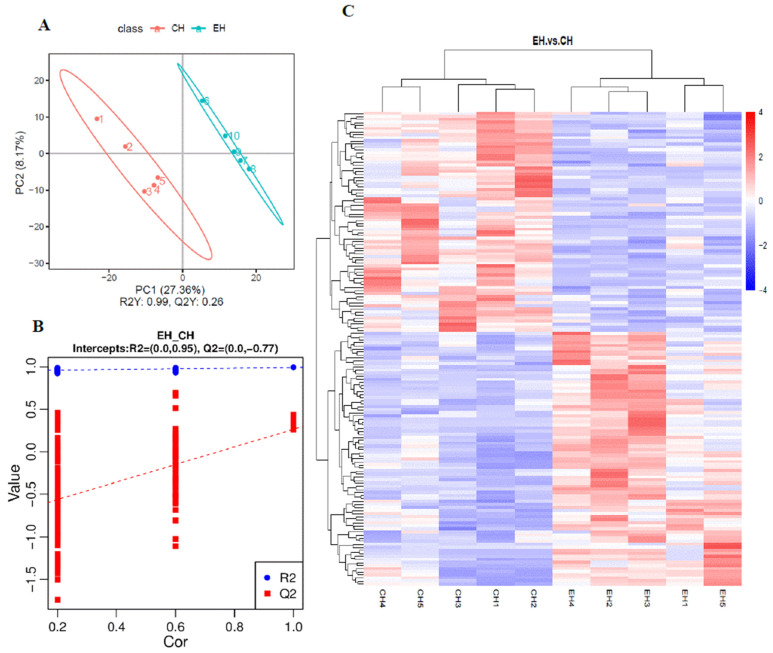

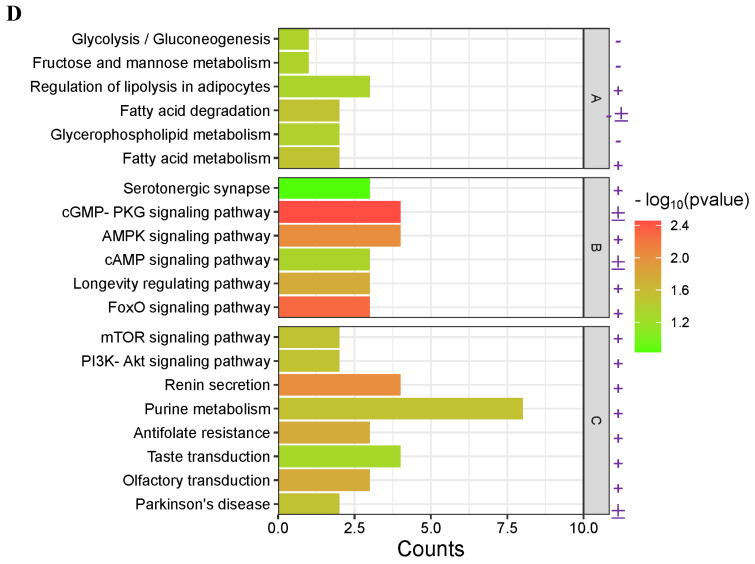
Metabolome changes in heart of *E. sinensis* under alkalinity stress. (**A**). Analysis of PLS-DA, CH represents the control group, EH represents the experimental group. (**B**). Permutation test of PLS-DA. (**C**). Heatmap of DEMs after alkalinity stress. CH1–CH5 represents the control group, EH1-EH5 represents the experimental group. Each row represents one differentially expressed metabolites. (**D**). Top20 KEGG enrichment analysis on DEMs after alkalinity stress. Letters “A–C” represent three functional categories that the pathways can be divided into: A, energy metabolism; B, signal transduction; C, homeostasis maintenance. “+” indicates upregulated pathways, “−” represents downregulated pathways, “±” indicates the pathways that enriched upregulated and downregulated DEMs.

The results of PLS-DA analysis are shown in Figure 3A,B. The control group and the alkalinity-stressed group were clearly distinguishable, suggesting a significant variation in metabolite profiles in response to alkalinity stress. The classification coefficient R2Y reached 0.99, indicating that the model could reflect 99% of the differences between the two groups, and the PLS-DA model was reliable. The permutation test on the model parameters R2 and Q2 were carried out, and the number of tests was set at 200. As shown in Figure 3B, R2 was higher than Q2 and Q2 < 0, which indicated no over-fitting. As shown in Figure 3C, a total of 155 DEMs were detected in heart after alkalinity stress, among which, 83 DEMs were upregulated and 72 DEMs were downregulated. As shown in Figure 3D, top20KEGG pathways mainly involved three aspects: energy metabolism, signal transmission, and organismal systems and their homeostasis maintenance. Energy-metabolism-relevant pathways mainly involved glucose and lipid metabolism, most of which were downregulated. Organismal systems pathways mainly included regulation on taste and smell systems. Homeostasis maintenance pathways mainly involved longevity regulation, antioxidant response, blood pressure regulation, and damage repair of DNA and RNA, most of which were upregulated. 

### 3.4. Integrated Analysis on Cerebral Ganglion Proteomics and Heart Metabolomics under Alkalinity Stress

**Figure 4:**
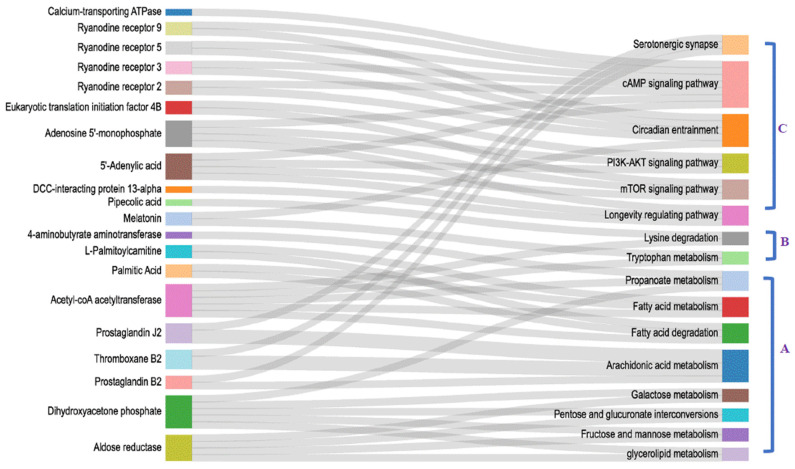
Co-regulatory pathways and corresponding DEPs/DEMs enriched from cerebral ganglion proteome and heart metabolome of *E. sinensis* under alkalinity stress. The right column indicates co-regulatory pathways. The left column shows the corresponding DEPs/DEMs. “A–C” represents three functional categories that the co-regulatory pathways can be divided into. A, energy metabolism; B, amino acid metabolism; C, homeostasis maintenance.

As shown in Figure 4, sixteen co-regulatory pathways were enriched, which can be classified into three categories: energy metabolism, amino acids metabolism, and antioxidant homeostasis maintenance. Among which, eight energy-metabolism-related pathways mainly involved glucose and lipid metabolism, and most of them were downregulated. Amino-acid-metabolism-relevant pathways mainly involve tryptophan and lysine metabolism. Pathways related to antioxidant homeostasis maintenance and signal transduction mainly involve regulation on anti-stress response and longevity.

## 4. Discussion

**Figure 5:**
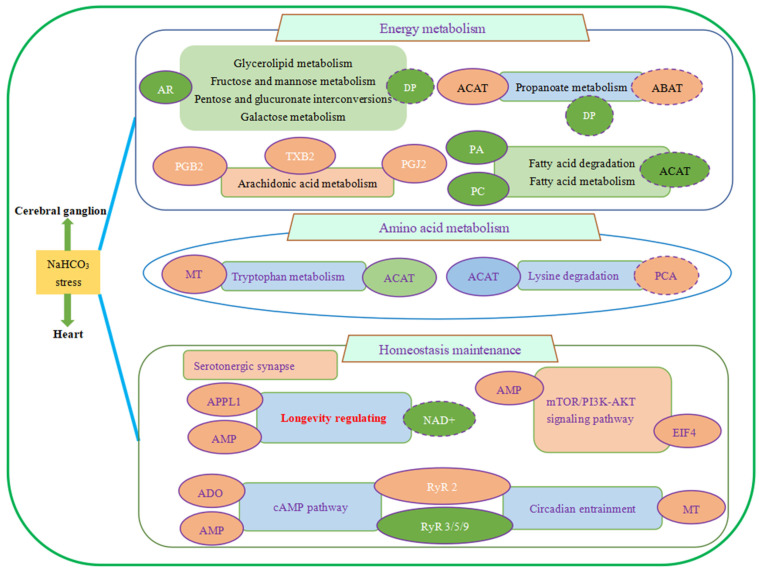
Schematic overview of co-regulation mechanism of cerebral ganglion and heart under alkalinity stress. The enriched pathways are marked with a rectangle; the DEPs/DEMs are marked with circles. Upregulated pathways and DEPs/DEMs are shown in red; downregulated pathways and DEPs/DEMs are shown in green; and the pathways that were both upregulated and downregulated are shown in blue. The DEPs/DEMs, which caused injury in this study, are indicated by a dotted line. Pathways with significant correlations are interlinked with arrows.

In this study, the key differential regulatory pathways and co-regulatory pathways of the cerebral ganglion and heart after alkalinity stress all mainly involve energy metabolism, amino acid metabolism, signal transduction, and antioxidant homeostasis maintenance, reflecting the close relation between these two tissues in response to alkalinity stress (Figure 5).

### 4.1. Regulation on Energy Metabolism and Signal Transduction in Cerebral Ganglion and Heart under Acute Alkalinity Stress

In this study, energy metabolism pathways were relevant to carbohydrate and lipid metabolism and most of them were downregulated, such as “Fructose and mannose metabolism”, “Pentose and glucuronate interconversions”, and “Galactose metabolism”, as well as regulatory molecules aldose reductase (AR) and dihydroxyacetone phosphate (DP). As a member of the aldosterone reductase family, AR has been extensively studied in human diseases. AR is upregulated in many osmotic and oxidative injury processes [25]. 

In this study, the expression of Thromboxane B2 (TXB2), Prostaglandin B2 (PGB2), and Prostaglandin J2(PGJ2) in the arachidonic acid signaling pathway were significantly upregulated. Osmotic stress studies on many aquatic species, such as the salinity adaptation mechanism of the nervous system in Clam (*Ruditapes philippinarum*) and the regulation mechanism of hepatopancreas in *E. sinensis* under saline–alkali conditions, have both indicated that the arachidonic acid pathway plays an important regulatory role in neurotransmitter transmission during osmotic stress [13,26]. In the present study, the expressions of this pathway are also significantly upregulated, which reflects its extensive regulatory role in various tissues in resistance to osmotic stress. As an important intermediate product of the arachidonic acid pathway, prostaglandins have multiple physiological functions, including causing gastrointestinal smooth muscle contraction to protect gastric mucosa and regulation on neurotransmitters’ release and blood pressure. As a prostaglandin derivative, TXB2 can induce platelet aggregation to cure tissue injury and inflammation [27]. In this study, its upregulation is also an active anti-inflammatory response in the heart of *E. sinensis* in resistance to environmental factor stress.

In addition, pathways relevant to fatty acid metabolism (fatty acid degradation, fatty acid metabolism) and regulatory molecules (Acetyl-coA acetyltransferase, Palmitic Acid and Palmitoylcarnitine) were downregulated. Studies on mice have shown that palmitic acid (PA) has a toxic effect on the heart [28]. In this study, its downregulation is also a positive anti-damage response of the heart in *E. sinensis* under alkalinity stress. Palmitoylcarnitine (PC) can also cause cardiotoxicity, including calcium homeostasis disturbance and mitochondrial energy failure [29]. In this study, its downregulation also reduces the possibility of myocardial dysfunction and is conducive to maintaining cardiac function.

### 4.2. The Co-Regulation of Cerebral Ganglion and Heart on Signal Transduction under Alkalinity Stress

In this study, alkalinity stress led to significant differential expression of some signal transduction pathways, such as tryptophan metabolism and lysine degradation. Serotonin is an important neurotransmitter. As the sole precursor of serotonin, tryptophan participates in the regulation of neuronal transduction [30]. Melatonin (MT) was upregulated in the tryptophan metabolism pathway after alkalinity stress. As an antioxidation regulator, MT plays a modulatory role in cardiovascular rhythm and protects heart activity, thus reducing the probability of heart disease [31]. Studies on plants have shown that MT plays an active regulatory role in ion homeostasis and antioxidation activity against alkali stress [32]. It has also been reported that MT can improve the resistance of *E. sinensis* to adverse environmental factors, such as hypoxia, and protect its immune system [33]. In this study, its upregulation is beneficial to protecting the cerebral ganglion and heart from injury under alkalinity stress. In addition, pipecolic acid (PCA) in lysine degradation is significantly upregulated. PCA is a common metabolite of lysine decomposition. It has been reported that the occurrence of metabolic disturbance in the central nervous system is always accompanied by an increase in the level of PA, which causes oxidative stress [34]. In view of the positive regulatory role of the downregulation of ABAT on neuronal signal transduction in these two pathways, the differential expression of these two pathways in this study reflects the positive regulatory role in anti-alkalinity stress.

### 4.3. Synergistic Regulation on Longevity and Antioxidant Homeostasis Maintenance under Alkalinity Stress

In the present study, the longevity regulating pathways and regulatory molecules, including DCC-interacting protein 13-alpha (APPL1), 5′-Adenylic acid (AMP), and NAD+, were differentially expressed after alkalinity stress. As a multifunctional adapter protein, DCC-interacting protein 13-alpha can combine with multiple membrane receptors, nuclear factors, and signaling proteins to mediate anti-inflammatory response and protect endothelial cells. It plays a key regulatory role in various processes, such as maintaining energy homeostasis, cell proliferation, and immune response and preventing atherosclerosis [35]. Adenylate, also known as adenosine-5′-monophosphate, is an important energy transmitter [36]. In this study, both of the above-mentioned regulatory molecules were upregulated after alkalinity stress to play a positive regulatory role in resistance to stress. As an important organ for dynamic regulation, the heart has been reported to be related to the life span of organisms [37]. In this study, the differential expression of the longevity regulation pathway in the heart and cerebral ganglion after alkalinity stress suggests that the cerebral ganglion and heart may be determining organs in the survival of *E. sinensis* under alkaline conditions.

SOD and GSH-Px are pivotal antioxidant enzymes that play a crucial role in scavenging oxygen radicals and maintaining cellular homeostasis [38]. T-AOC is an important antioxidant capacity indicator. In the present study, the results demonstrated that acute alkalinity stress for 6 h induces an upregulation of antioxidant levels in *E. sinensis*, thereby facilitating an anti-stress response in *E. sinensis*. Antioxidant response regulation is closely related to ageing. In this study, some pathways related to antioxidant response and aging (phosphatidylinositol 3-kinase—serine/threonine kinase AKT (PI3-AKT), and mTOR pathways) were also significantly upregulated. The PI3K-AKT signaling pathway can regulate cell proliferation, differentiation, and key antioxidant enzymes in response to extracellular signals. The research on the response mechanism of black tiger shrimp *Penaeus monodon* and Hilsa shad (*Tenualosa ilisha*) under salinity stress have both showed that it plays an important role in antioxidant defense [39,40]. As a central regulator of longevity and environmental adaptation, mTOR plays a regulatory role in many physiological processes related to aging, including autophagy and antioxidant homeostasis [41]. In this study, in addition to the upregulation of 5′-Adenylic acid, Eukaryotic translation initiation factor 4B (EIF4B) was also significantly upregulated in both pathways. Studies on human disease have shown that a reduction in mTOR and EIF4B have adverse consequences on synaptic function and may affect the function of the nervous system [42]. In this study, its upregulation is a reflection of a positive response of the cerebral ganglion and heart in *E. sinensis* against alkalinity stress.

As a second messenger, cAMP plays a regulatory role in calcium homeostasis [43]. In this study, except for the upregulation of 5′-Adenylic acid and adenosine on the cAMP pathway, ryanodine receptor 2/3/5/9 (RyR2/3/5/9) also exhibited differential expression. As an endogenous nucleoside, adenosine (ADO) can generate adenylate via phosphorylation and participate in myocardial energy metabolism [44]. As a channel protein, the ryanodine receptor plays a regulatory role in the release of intracellular calcium ions and muscle movement. Precise regulation of RyR2 activity is essential for each heartbeat [45]. In this study, RyR2 was upregulated and RyR 3/5/9 were downregulated to jointly play a positive regulatory role in cardiac activity in response to alkalinity stress.

As an important neurotransmitter, serotonin participates in regulation in terms of feeding, body temperature, movement, and reproduction. It has been reported that serotonin in the brain is closely coupled with the arachidonic acid pathway via phospholipase A [46]. In this study, the above-mentioned two pathways enriched the same DEMs and were both upregulated at the same time after alkalinity stress, which indicates the regulatory consistency of them in response to alkalinity stress.

Circadian entrainment synchronizes an organism’s endocrine and behavioral rhythms with environmental signals [47]. MT is a hormone produced in the pineal gland in the brain, which plays a crucial role in the circadian rhythm maintenance of an organism [48]. In this study, its upregulation is conducive to homeostasis maintenance in response to alkalinity stress.

## 5. Conclusions

This study is the first to investigate the synergistic regulatory mechanism of the cerebral ganglion and heart in *E. sinensis* under alkalinity stress. The results show that these two organs have a close relation in response to alkalinity stress. The co-regulatory pathways mainly involve regulation on energy metabolism, amino acid metabolism, and homeostasis maintenance. Importantly, alkalinity stress caused regulation of the cerebral ganglion and heart on antioxidant response and further on longevity, suggesting that the cerebral ganglion and heart may be the determining tissues for the survival of *E. sinensis* under an alkalinity environment. In the future, further investigation into functional regulators specific to the cerebral ganglion and heart may be carried out to supplement the relevant research on the enhancement of the anti-stress response ability of these two tissues. This study provides a theoretical reference for the research on the regulation mechanism of *E. sinensis* under saline–alkali conditions; it also provides a theoretical guidance on the cultivation of new varieties of saline–alkali-tolerant *E. sinensis* and contributes to the development of aquaculture in saline–alkali water.

## Data Availability

Raw data were submitted to BIG Sub Database (PRJCA027286). All other data are contained within the main manuscript.
